# “Of Sheep and Men”: Earliest Direct Evidence of Caprine Domestication in Southern Africa at Leopard Cave (Erongo, Namibia)

**DOI:** 10.1371/journal.pone.0040340

**Published:** 2012-07-11

**Authors:** David Pleurdeau, Emma Imalwa, Florent Détroit, Joséphine Lesur, Anzel Veldman, Jean-Jacques Bahain, Eugène Marais

**Affiliations:** 1 Département de Préhistoire, Muséum National d'Histoire Naturelle, UMR 7194 du CNRS, Paris, France; 2 National Museum of Namibia, Windhoek, Namibia; 3 Département Écologie et Gestion de la Biodiversité, Muséum National d'Histoire Naturelle, UMR 7209 du CNRS, Paris, France; University College London, United Kingdom

## Abstract

The origins of herding practices in southern Africa remain controversial. The first appearance of domesticated caprines in the subcontinent is thought to be c. 2000 years BP; however, the origin of this cultural development is still widely debated. Recent genetic analyses support the long-standing hypothesis of herder migration from the north, while other researchers have argued for a cultural diffusion hypothesis where the spread of herding practices took place without necessarily implicating simultaneous and large population movements. Here we document the Later Stone Age (LSA) site of Leopard Cave (Erongo, Namibia), which contains confirmed caprine remains, from which we infer that domesticates were present in the southern African region as early as the end of the first millennium BC. These remains predate the first evidence of domesticates previously recorded for the subcontinent. This discovery sheds new light on the emergence of herding practices in southern Africa, and also on the possible southward routes used by caprines along the western Atlantic coast.

## Introduction

Southern Africa possesses a rich diversity of human populations that can be differentiated by genetic [1]–[5], linguistic [6]–[10] and subsistence strategies [7]. The origin and history of this peopling process is the focus of numerous debates, including the possible origin of *Homo sapien*s and modern human behaviour (see for instance [11]–[13].

During the last three millennia in particular, several waves of migration occurred southwards towards the subcontinent. This is the place of origin for the San people, a unique group of hunter-gatherer lineages belonging to the oldest known modern human populations [2], [5]. The later migrations of agro-pastoralist Bantu (about ∼1500 years ago) and the more recent arrival of first Europeans are well-documented. However, the origins of the earliest herding practices are far more problematic and beset by difficulties in combining studies of various types (linguistic, archaeological, genetic and so forth) [14].

The oldest evidence for domesticated animals in southern Africa is generally accepted to be around c. 2000 BP. Although direct dating of these sites is rare, several sheep bones have been recovered in Later Stone Age (LSA) contexts (late first millennium BC/early first millennium AD). This date (or perhaps slightly earlier) may also correspond to significant technological changes, including the first appearance of pottery [18]–[21]. This time period is therefore a key stage in the subsistence economy of hunter-gatherer groups in southern Africa.

The region also experienced significant climatic and environmental changes over this time period, with a drying period succeeding a wetter period. The latter could have been conducive to the migration of animals through the presently very dry Kalahari area. This area may have acted as a corridor of migration (for both humans and animals) c. 2500–2000 ([22] and see [18], [23]).

The main routes of southward migration are as follows: a central route through the Kalahari and the Orange River [24]–[27] or an alternative migration route that runs parallel to the western coast of Namibia [9], [28]–[31]. Namibia lies at the centre of both routes, making it a potentially rich source of information about these migrations. Some Namibian sites, such as Geduld [32], Mirabib [33]–[35] or Oruwanje 95/1 [19] have already yielded early remains of domestic animals (caprine bones or dung layers). However, the sheep from Geduld and Mirabib and the goat from Oruwanje 95/1 lack direct dates.

Although sheep or goats migrated to southern Africa around 2000 BP, this does not necessarily mean that they were accompanied by an influx of new human migrants. For over a century, many researchers (linguists, anthropologists, archaeologists) have favoured the demic hypothesis to explain the arrival of domesticates i.e. the arrival of significant “proto-Khoekhoe” groups, hypothetical ancestral populations of the pastoralists “Hottentots” (Khoe speaking) encountered by the first Europeans who arrived on the southernmost coast of Africa from the end of 15th century onwards [18], [21], [24], [28], [29], [36]. Several recent genetic and linguistic studies support this hypothesis, although the place of origin of these early herders and/or stock is still widely debated [4], [6], [37]–[41].

The demic hypothesis is poorly supported archaeologically because of a lack of evidence. A few isolated domesticate bones from LSA hunter-gatherer contexts from around 2000 BP have been recovered [42], and no physical anthropology study allows suggesting an arrival of new population (partly complicated by a lack of human remains) [43], [44]. Alternatively, cultural diffusion may have occurred; with animal domestication gradually introduced into local hunter-gatherer groups by cultural percolation with northern herders (“hunters with sheep” [25]), without invoking a complete social change [25], [45], [46].

Within this context, we here present our excavation results from the Holocene site of Leopard Cave (Erongo Mountains, Central West Namibia) discovered in 2006 by a French-Namibian team from the National Museums of Paris and Windhoek. Leopard Cave documents a succession of LSA human occupation layers from 3200 BP onwards, which have yielded several hundred artefacts and faunal remains, including directly-dated caprine remains. This is the earliest evidence of domesticates in southern Africa, and it adds to the debate regarding the possible timing and routes of domesticated animal migration towards the southernmost of Africa between approximately 2000–2500 years ago.

## Materials and Methods

**Figure 1:**
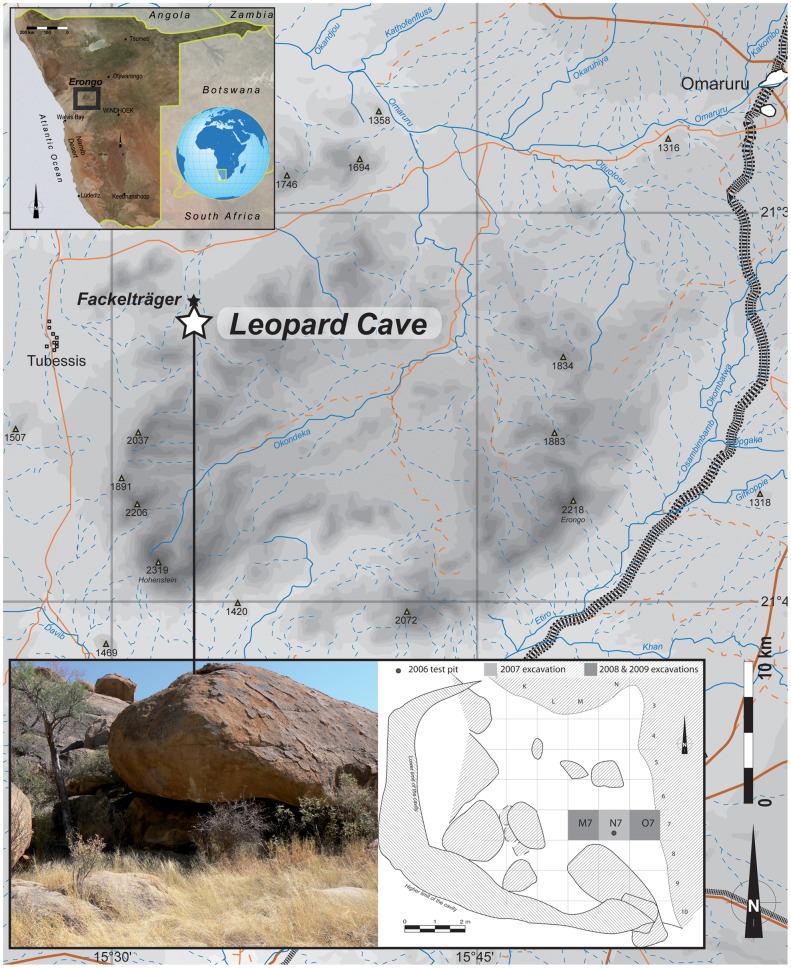
Location of Leopard Cave (Omandumba West, Erongo, Namibia). Plan of the cave and location of the excavated area.

Leopard Cave is located on the farm of Omandumba West (S 21°34′22″; E 15°33′18″) in the vicinity of Omaruru, northwest Erongo Region (∼200 km NW of Windhoek) (Figure 1). It is located ∼1.7 km south of the LSA site of Fackelträger [47], ∼25 km south of the Etremba sites [47], [48] and ∼25 km to the north-east of the Big Elephant and Striped Giraffe sites [49], which also contain rich LSA sediments. The Erongo area is renowned for providing a rich archaeological record starting from the early Middle Pleistocene [50]. Leopard Cave is a rock shelter of about 50 m^2^ (∼ 7 meters a side), of which about half is covered with granite boulders (Figure 1). It lies at an altitude of 1256 meters (asl) at the bottom of a cretaceous granite massif to the north, directly facing the northern hills of the Erongo Mountains. The site was discovered in 2006 during an archaeological survey. The majority of the excavation took place during December 2007, thereafter sporadically in 2008 and 2009. Until now, 3m^3^ of sediments have been excavated from a 3×1 metre trench (squares M7, N7 and O7) (Figure 1). The substratum has not been reached and a test core indicates that at least 50cm of artefact-bearing sediment remains unexcavated.

**Figure 2:**
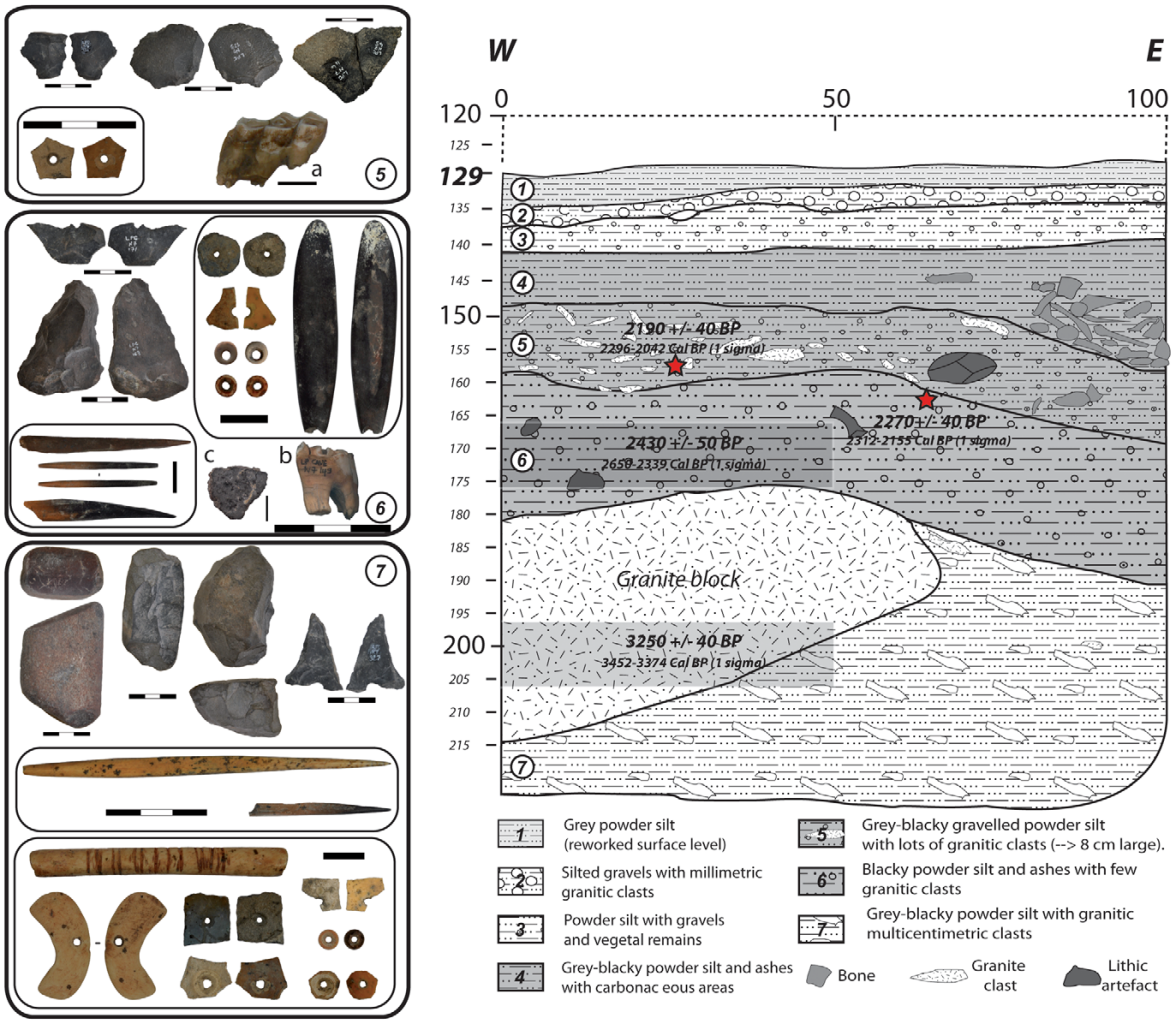
Synthetic stratigraphic section E-W (transversal) of 7/8 band of Leopard Cave (in cm), with location of the dated samples. Examples of archaeological remains found in Layers 5, 6 and 7.

We excavated in 10cm spits (within subsquares of 0.5 m side), with constant evaluation of the lithostratigraphic conditions (changes in colour and texture). All archaeological remains (>2 cm in size) were plotted in a 3D grid system, and all excavated sediment was dry-sieved (through a 2 mm mesh). Seven sub-horizontal mix layers of granitic “arena” with a silt texture have been identified, numbered from one (the youngest) to seven (current base of the sequence) (Figure 2). The texture is due to the powdered silts resulting from the breakdown or chemical alteration of the granite boulders. The arena is in a large part mixed with ashes of anthropogenic origin. Some of the layers also contain clastic residues of granite, of varying size.

## Results

The archaeological layers contain lithics, charcoal and faunal remains (including beads and bone tools). Small potsherds were also found in Layers 4, 5 and 6. After sieving all excavated sediment, more than 4600 specimens have been recovered, including small lithics (chips), ostrich eggshells and small bone fragments.

### Lithic artefacts

The lithic artefacts (n = 2651) are almost exclusively produced from local basalt (71%) and quartz (26%) throughout the sequence. The outcrops of basalts flows and quartz dykes are located in the Erongo pluton, where Cretaceous volcanic flows (rhyolith, basalt) overlie the Palaeozoic and lower Cretaceous granites. Few cortical residues are visible on the quartz material, and the rolled aspect of the basalt artefact cortex suggests that the basalt could have been collected from secondary sources, in the alluvial deposits of the area (i.e. river cobbles). Only one piece of chert was found in the entire sequence, from Layer 5. Chert is not found in the Erongo region and the closest source is located in the limestone and marble formation of the Damara Orogen, with the nearest outcrop located between 60 to 80km from the site [51]. This single artefact made on an exotic raw material provides little information about raw material procurement strategies, but it may suggest that the inhabitants enjoyed greater mobility during this time.

The artefacts are generally in an excellent condition, with only fire-related surface alterations. Approximately 100 pieces (excluding small fragments), made mainly on basalt, show fire damage in the form of rubefied zones, cracks and fractures. Most of the fragments are too damaged to be analysed further. This fire damage appears to be associated with the hearths that can be seen throughout the stratigraphic sequence.

The technical behaviours associated with the artefacts are difficult to infer at present. The lithic assemblage contains very few retouched artefacts and no formal tools. Certain large basalt flakes show retouching, generally on both sides. We infer that part of the knapping process took place *in situ*, based on the presence of some cores and thousands of small chips (small flakes and debris) recovered in the cave. Directly in front of the cave, lithic artefacts covering a large surface area (more than 100m^2^) have been recovered and test pits are planned to assess the relationship between the cave occupations and this area.

No clear technical variation can be observed throughout the sequence. When chips and small debris are excluded (n = 2191), the assemblage is mainly composed of non-diagnostic flakes (62%). There is also a high proportion of cobble fragments (35% of the non-waste material), largely due to fire damage. The average flake size (∼35 mm in length) does not vary with the raw material used. The flakes show few dorsal scars, which indicate an almost exclusive use of a unidirectional reduction method. Twenty-eight cores (n = 28) have been recovered, mostly fragmented. Only ten cores retain sufficient visible flake scars to track the technological processes. Among them, several split oval pebbles (n = 7) have been found in layers 5, 6 and 7. They show several unipolar removals from the fracture and resemble heavy tools (*nucléus-rabot* like) (Figure 2, Layer 7). Certain of these appear to have been used as hammerstones, with some percussion marks (crushing, wrenching and/or typical fracture) still visible on one, or both, rounded ends. Another split cobble appears to be the result of a bipolar technique on anvil (showing two opposite impact points). There are only three exhausted cores made on quartz (length <25mm). Their reduction involved two convex debitage surfaces, alternatively and recurrently flaked (radial flaking).

Two flaking techniques have been used. The use of a direct percussion with a hard hammer is indicated by the presence of certain pebbles with hammerstone scars (crushed areas on their edges), and by the large and flat striking platform and the marked bulb visible on the majority of the flakes (especially basalt ones). The bipolar technique is also indicated by some double opposite impacts points and cones (principally on quartz). We are presently conducting experimental studies to establish whether or not specific granite slabs were used as anvils.

Lastly, one basalt grinding stone was recovered from Layer 7 (Figure 2, Layer 7). The grinding stone shows a strong abraded surface on one of its extremities, where some pigment residues are still visible. In addition, this artefact appears to have been used as a hammerstone, since the opposite end shows clear signs of crushing.

### Non-lithic artefacts

The non-lithic artefacts recovered include bone tools, as well as a few small potsherds. Also recovered are beads and pendants made of ostrich (*Struthio camelus*) eggshell and animal bones.

#### Bone tools

A sample of ten bones, all from Layers 6 and 7, show working as bone points (Figure 2). The most frequent bone tool (n = 8) is a sharp point, which is either partially burned (at the tip) or completely burned (n = 3). Two slender linkshafts – one complete and one broken at the base – have also been recovered. These points are comparable to bone tools described from the neighbouring sites of Fackelträger [47], or Big Elephant Shelter [49] and more generally in LSA (and even MSA) sites of southern Africa (see [52]). Particularly, the linkshafts are consistent with the Clark's 1975 ethnographic classification of Bushmen arrows used in southern Africa, in particular, of arrowheads type 3 or 4 [53]. Some discoveries in South Africa have pushed back the appearance of complex bone technologies (even sporadically) to ca. 60–80 ka in Middle Stone Age (MSA) occupations, like in Blombos Cave [54] or Sibudu [52]. These bone tools types are less common in the MSA, whereas they are commonly recovered during the LSA.

Several worked bone shafts show signs of polishing. However the original tool form is difficult to determine, given the damaged state of these pieces.

Amongst the worked bones, we have identified an avian long bone with signs of polish, as well as multiple transverse deep incisions covered with red pigment (Figure 2, Layer 7). This find is unusual, and so far, no similarly marked objects have been reported from any other Namibian LSA site.

The bone used for the tools cannot be identified to species, but we can state that all but one of the bone tools were made from mammalian long bones, while the final tool is made on the bird bone mentioned previously. This pattern may potentially reveal aspects of the hunting strategies and techniques (such as the preferential use of bone-tipped projectiles) represented in Layers 6 and 7 of Leopard Cave. Ethnographical comparisons made on some bone tools from MSA and LSA sites indicate a strong link with the bone points used by hunter-gatherers Bushmen (with or without poison) until at least the 19th century [52].

#### Ornamentation: Beads and pendants

Ostrich eggshells have been abundantly recovered throughout the sequence. Thirty-two ostrich eggshell beads (OEB) and four pendants (two broken ostrich eggshell pendants and two complete bone pendants) were excavated from Layers 4, 6 and 7. The pendants were recovered from layer 6 (n = 3) and 7 (n = 1) (Figure 2). The OES pendants show drilling (from the concave side) and subsequent polishing on both sides (principally visible on their edges for the OES pendants). One OEB pendant now lacks the section with the aperture. The pendants are of three shape categories oblong (47×10×2 mm) and crescent (26×8×1.5 mm) for the bone pendants; rounded for the broken OES pieces (one circular and one oval). Both bone pendants show irregular striations on both faces.

The sample of thirty-two OES beads (deriving from Layer 4, n = 1; Layer 6, n = 16; Layer 7, n = 15; Table 1) represent three different stages of manufacture (and even potentially different *chaînes opératoires*) [55], [56]. One involved the initial trimming of the OES blank. The majority of these unfinished specimens (n = 10) have raw broken sides. Typically, these blanks have four sides, although two pieces are pentagonal. The pentagonal beads are the largest, measuring between 6.5 mm to 12.5 mm a side. Two of them are also fractured around the aperture, suggesting that drilling caused these fractures.

**Table 1:** External and aperture diameters, and thickness of finished beads of Leopard cave (in mm).

bead diameter		aperture diameter		thickness	
Layer 6		Layer 6		Layer 6	
n	11	n	11	n	11
mean	4,73	mean	1,56	mean	1,42
min	4	min	1	min	1,1
max	5,2	max	2	max	1,7
sd	0,38	sd	0,28	sd	0,18
Layer 7		Layer 7		Layer 7	
n	8	n	8	n	8
mean	4,74	mean	1,45	mean	1,58
min	4,2	min	1,3	min	1,4
max	5,4	max	1,9	max	1,7
sd	0,43	sd	0,19	sd	0,13

mean aperture of unfinished beads (n = 13) = 1,43mm.

The second kind of bead manufacture (n = 19) indicates the finished stage of the bead making, meaning that the drilling phase (mostly from the both sides of the eggshell) was followed by polishing. The rounded shape and the size variables (diameter, aperture diameter and thickness) of these beads show little variation within each layer and no significant differences between Layer 7 to Layer 6 (Table 1). All the beads are smaller than 5.4 mm in diameter (mean = 4.7 mm), with aperture diameters smaller than 2 mm (mean = 1.5 mm).

Finally, we have identified a third kind of bead manufacture, which is intermediate between the two techniques discussed above. Indeed, the shape of three drilled OES is already rounded (bead diameter ranging from 7.5 to 9.4mm), but raw edges remain visible and the beads lack polish.

These types of pendants, and in different stages of the manufacture of OEB, have already been reported from other sites in the Erongo area, like Fäckeltrager [47] and the Big Elephant Shelter [49]. Particularly, OES beads have been recovered from a number of LSA sites along the Atlantic coast, from Central Namibia to Namaqualand and the Northern Cape, and the issues concerning their size variation through time are strongly debated [20], [32], [56]–[63]. Certain researchers support the hypothesis that the overall size of OES beads can be used to distinguish LSA sites as either hunter-gatherer or herder-pastoralists in origin [32], [57], [59], [64].

According to Smith and colleagues' model [59], hunter groups (“Bushmen”) preferred small beads (diameter <5mm), while herder groups (“Khoekoe”) preferred larger ones (>6mm). Their conclusion was based on a number of sites in the southwestern Cape, including Kasteelberg. At Leopard Cave, no large (>6mm) or very large (also called “discs” by Wendt [47]) (>10 mm) finished beads have been recovered. Following this model, that would suggest an occupation by hunters in layers 6 and 7, although the beads do co-occur with the first caprine remains. However, Kinahan [60], [65] challenged the bead size hypothesis of Smith and colleagues [59], [66]. More recently, re-examining beads recovered from several sites in Kasteelberg, Sadr and al. [62] consider this size difference “to reflect change through time rather than representing emblems of different but contemporary cultures”.

#### Pottery

Five pottery sherds have been recovered in in same square (N7). Apart from one sherd, which was found in Layer 6 (just below the first caprines remains), all the others come from Layer 4 (n = 2) and from limits between Layers 5 and 4 (n = 2), where several other caprines remains have been found. These undecorated and non-diagnostic body sherds can be refitted into pairs (Figure 2, Layer 5). They presumably derive from two different pots, as they differ in their thickness (18–12mm and 5 mm). The sherd from Layer 6 is small (25×22×4 mm), thin (4 mm), well-fired and undecorated (Figure 2, Layer 6), which is consistent with the first ceramics, which appeared in the subcontinent in the last centuries BC [67], [68].

### Faunal assemblage and the domesticate evidence

A total of 1811 faunal remains have been discovered to date (2007–2009), of which 406 (∼22%) can be identified to various taxonomic levels (class, family or species). The low percentage of identification can be partly explained by the heavy fragmentation of the bones, with 60% of the assemblage less than 2cm in maximum length. Identification to taxonomic level and identification of human modification is further complicated by post-depositional processes such as erosion, weathering and root etching. However, some long bones present spiral-shaped fractures characteristic of breakage on fresh bones that could indicate the consumption of marrow by the inhabitants of the site and/or the preliminary stages for bone tool manufacture. Associated with the rest of the archaeological material, this evidence confirms that humans accumulated the faunal assemblage and, although cut marks are very few due to significant levels of post-depositional damages. Although cutmarks are very few, they suggest, together with burnt bones and fresh fractures, that this assemblage was accumulated as consumption refuse.

The detailed faunal analysis can be compared to other sites to provide new information on the lifestyles of people in the Erongo Region during the Holocene. The assemblage is homogeneous throughout the entire sequence and no difference in faunal composition can be observed over time. Therefore we present and discuss the detailed results coming from Layers 5 and 6 as representative of the overall sequence. The complete assemblage will be published in the near future.

Of the 866 remains found in Layers 5 and 6, we were able to identify 237 fragments, including 144 fragments of ostrich eggshells (Table 2). Bird species such as the helmeted guinaefowl (*Numida meleagris*) and reptiles such as the monitor lizard (*Varanus niloticus*) and tortoise (Testudinae indet.) are represented. Mammals are the most commonly recovered taxonomic group, especially small to medium-sized bovids, including several specimens of wild species such as impala (*Aepyceros melampus*) and klipspringer (*Oreotragus oreotragus*). All taxa are represented by a minimum number of one or two individuals.

**Table 2:** Faunal list of the squares N7, M7 and O7 (layers 5 and 6) of Leopard Cave.

	NISP
Bovidae size 3	5
Bovidae size 2	11
Sheep/goat (Caprinae)	2
Springbuck *(Antidorcas marsupialis)*	1
Bovidae size 1	4
Klipspringer *(Oreotragus oreotragus)*	1
Rock hyrax *(Procavia sp.)*	4
Rodent sp. indet.	16
**Total Mammals**	**44**
Bird sp. indet.	20
Helmeted guineafowl (*Numida meleagris*)	1
**Total Birds**	**21**
reptile sp. indet	12
Monitor lizard *(Varanus niloticus)*	6
Tortoise (Testutinidae sp. indet.)	10
**Total Reptiles**	**28**
Ostrich eggshells (*Struthio camelus*)	144
*Unidentified*	*629*
**TOTAL**	**866**

The rest of the spectrum includes rock hyrax (*Procavia capensis*) and rodents. For the latter, the very fresh appearance of the bone surfaces suggests that they are intrusive and were deposited after the human occupation of the site. In spite of its small size, the bone assemblage indicates that people relied mainly on medium-sized bovids for meat supply. Moreover, the presence of impala, klipspringer and rock hyrax, all mixed feeders, suggests that conditions were similar to those existing today; with grassy, and rocky hills interspersed with wooded patches.

**Figure 3:**
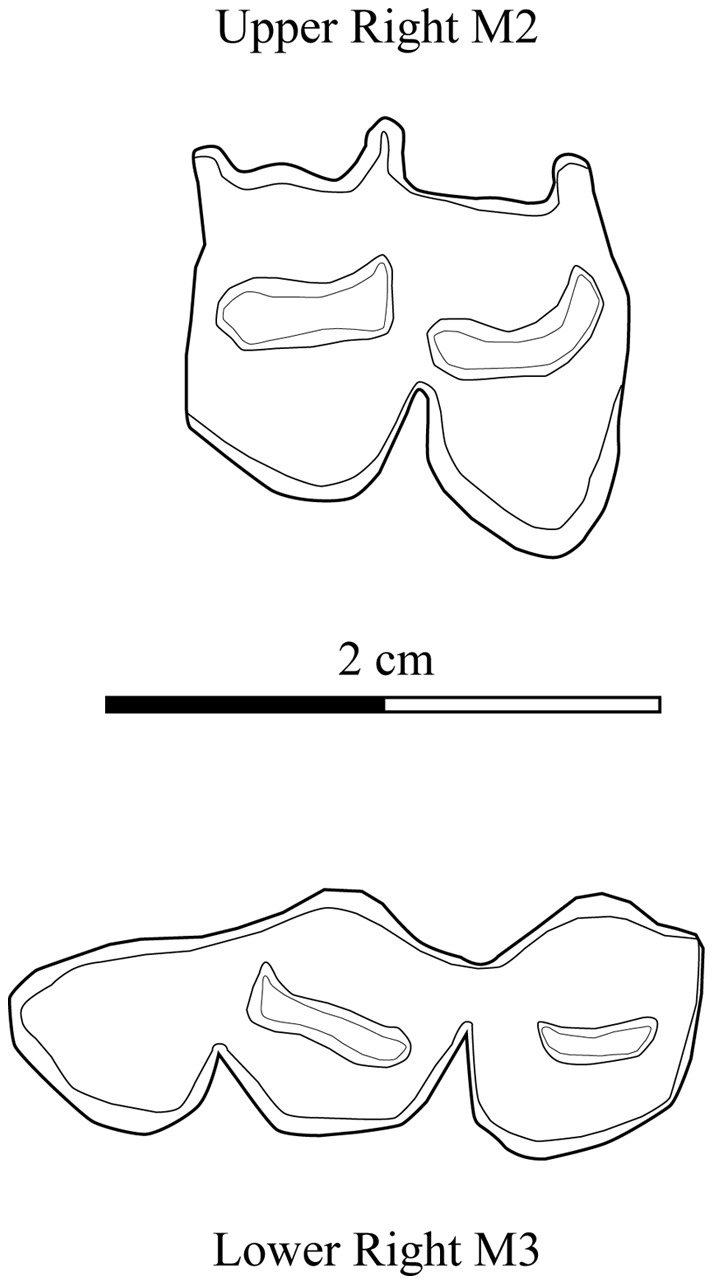
Cheek teeth from sheep/goat at Leopard Cave (Sq. N7, Layers 5 & 6).

Within the bovid sample, we found two caprine molars (Figure 3 and Figure 2, Layers 5 and 6). These teeth are characteristic of the Caprini, described as follows: “They (*Caprini*) have hypsodont cheek teeth without basal pillars, rather flat lateral walls between the styles of the upper molars, lower molars often with goat folds ….” [69]. Today there are no wild caprine species in southern Africa, although it appears that they existed in the southern Cape area during the Pleistocene and early Holocene. Reexamination of assemblages from Nooitgedacht 1 and 3 and Bloomplaas Cave in the Cango Valley by J.S. Brink has highlighted the presence of Caprini species, which presumably became extinct during the Holocene [70]. These wild caprines were very large and there is no evidence of their presence in the western part of the continent during the Holocene [25].

The size of the caprine teeth of Leopard Cave (Figure 3 and Table 3) is consistent with the modern collection of African domestic sheep (*Ovis aries*) and goats (*Capra hircus*) sampled in the collections of the *Muséum national d'histoire naturelle* (Paris, France). Therefore, there is no doubt that these molars derive from domestic caprines, but unfortunately the advanced tooth wear does not allow us to distinguish between sheep or goats. However, the overlying layer (Layer 4) has also yielded 13 caprine bones (mainly extremities of bones such as the talus, metatarsus and phalanges) that can be attributed to sheep (*Ovis aries*) suggesting that the dental caprine remains are very likely from a sheep also.

**Table 3:** Measurements (in mm) of the caprines teeth from Leopard Cave (A: Upper M2; B: Lower M3) compared with modern african specimens from the Muséum National d'Histoire Naturelle (MNHN).

	Origin	Species	N		Length	Width
A	Leopard Cave	*Ovis aries/Capra hircus*	1		15,6	16
	MNHN	*Ovis aries*	11	min.	14	14,5
				max.	18,1	19,4
				mean	16,3	16,8
	MNHN	*Capra hircus*	9	min.	13,9	14,3
				max.	17,6	18,7
				mean	15,7	15,9

	Origin	Species	N		Length	Width
B	Leopard Cave	*Ovis aries/Capra hircus*	1		25	9,7
	MNHN	*Ovis aries*	12	min.	21	7,9
				max.	27,1	10,8
				mean	24,8	9,1
	MNHN	*Capra hircus*	9	min.	20,3	7,5
				max.	26	10
				mean	24,2	8,6

N: Number of specimens; min: minimum; max: maximum.

### Dating

Accelerator Mass Spectrometry (AMS) radiocarbon dating was performed for four charcoal samples associated with the different layers from square N7 (Table 4). The radiocarbon dates have been calibrated, using the SHCal04 calibration curve, recommended for terrestrial material up to 11ka BP in the Southern Hemisphere [71]. Two dates taken during the 2007 excavation indicate episodes of human occupation around 2430±50 ^14^C BP (2650 to 2339 Cal BP) for Layer 6 and 3250±40 ^14^C BP (3452 to 3374 Cal BP) for Layer 7. A further date of 3180±40 ^14^C BP (3399 to 3266 Cal BP) was established from a charcoal sample recovered from the 2006 drill test, in a lower position than the current base of the excavation. Its error range overlaps at 1-sigma with the Layer 7 sample (Table 4). In addition to these dates, we have also directly dated the two Caprinae teeth unearthed from the Layers 5 and 6. Their radiocarbon ages just overlap, respectively 2190±40 ^14^C BP (2296 to 2042 Cal BP) and 2270±40 ^14^C BP (2312 to 2155 Cal BP).

**Table 4:** ^14^C dates at Leopard cave (calibration curve: SHCal 04 [71], using OxCal 4.1).

Lab#	Square	Layer	Depth (cm)	Nature	Conventional Radiocarbon Age BP	Age Cal BP1 sigma
Beta –270163	N7a	5	157,5	Tooth	2190 ^+^/_−_40	2169±127
Beta –270164	N7b	6	163	Tooth	2270 ^+^/_−_40	2233±78
Beta –236963	N7a	6	166–176	Charcoal	2430^+^/_−_50	2495±156
Beta –236964	N7a	7	196–206	Charcoal	3250 ^+^/_−_40	3413±39
Beta –236966	N7	?	254–279	Charcoal	3180 ^+^/_−_40	3333±67

All these dates are consistent with the overall site stratigraphy, suggesting that the dates obtained on the caprine teeth and charcoal from Layers 5 and 6 fit well with the archaeological framework. These teeth are therefore the earliest directly-dated caprines in southern Africa to date.

## Discussion

The Leopard Cave sediments have produced hundreds of LSA lithic artefacts and faunal remains as well as worked bone pieces and OES beads. The variety of remains throughout the sequence suggests that the occupations of the shelter were not directed towards specific types of activities (such as hunting, butchering or tool production). The significant fragmentation of the archaeological remains, especially of the fauna, and the amount of combustion by-products (such as ash, charcoal, burnt bone and lithics) indicate periods of intense human activity. The two caprine teeth dated to 2190±40 BP (2296–2042 cal BP) and 2270±40 BP (2312–2155 cal BP) have been respectively recovered from Layers 5 and 6 which are particularly rich in lithics, and which are also characterised by the absence of microlithic tools and abundant wild animal bones. The faunal analysis suggests that the inhabitants were hunting and consuming wild meat, such as birds, reptiles and antelopes. The presence of a few caprine bones and teeth from Layer 6 onwards indicates a limited exploitation of domesticate species, probably sheep. This scenario of inhabitants who mainly hunted wild game, but who also had some access to some sheep or goats, has been documented from other slightly younger sites in southern Africa [46].

**Figure 4:**
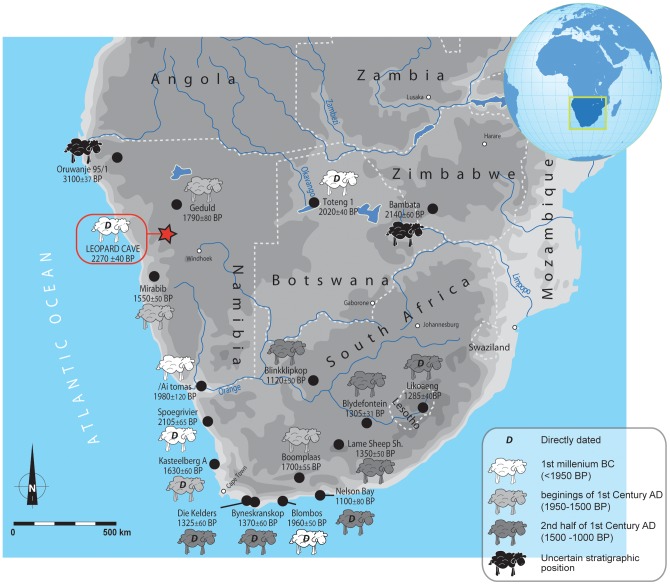
Later Stone Age sites of southern Africa with early evidence of caprines (after [**15]**–[17], [19], [30], [34], [57], [72], [83]–[87]).

The two directly-dated caprine teeth fit well within the stratigraphic context and ages. They are thus the oldest known remains of domesticates in Namibia and throughout southern Africa (Figure 4), as they predate the directly dated remains from the South African sites of Spoegrivier (2105±65 BP) [16], Blombos (1960±50 BP) [15] and Kasteelberg (1630±60 BP) [16], and the all in South Africa or from the Botswanan site of Toteng (2020±40 and 2070±40 BP) [17], [72]. Several sites which provided old dates (i.e. *c.* 2 ka), such as Oruwanje 95/1, in Namibia [19], Bambata in Zimbabwe [73] or Spoegrivier Layer 10 [31] for layers containing domesticates have been reported, but the chronostratigraphical position of the domesticate remains are questionable, since they were not directly dated (such as the possible down-section migration of remains, see [16], [31]).

The antiquity of the Leopard Cave caprines could support the hypothesis of southwards migration of domesticates following a route parallel to the west coast [16], [28]–[31], [74]. Furthermore, this time period corresponds to the end of a relatively humid period, which could have favoured the migration of domestic animals [22], [75] along this route to reach the Orange River, Namaqualand and southernmost Africa.

However, these sites where evidence of early domesticates (i.e. c. 2000 BP) have been found cover a large surface area and a short period of time (i.e. Toteng, Spoegrivier, Blombos and now Leopard Cave) (Figure 4), suggesting that migration routes must be interpreted with caution.

Migration routes are also often interpreted by means of the pottery sherd evidence, even if the link between the domestic package and pottery is still debated. Moreover, at this time period, there is still no clear evidence for discussing human migrations using ceramic stylistic variation [14], [76].

In the Namibian archaeological record (sites including Rock Falls shelter, Snake Rock and Geduld), Kinahan has argued for a gap between the appearance of ceramics and domesticates (∼1000 years later) [20] (and see discussions [32], [60], [61]). Layer 6 of Leopard Cave contained a piece of pottery in association with the early caprine remains. Although only a single undated fragment of pottery has been found so far, the present evidence would argue against Kinahan's hypothesis.

In any event, the Leopard Cave evidence cannot confirm whether or not the appearance of these pastoral elements (pottery and domestic) proceeds from the arrival of new human migrants. Recent genetic analyses of the inhabitants of southern Africa has confirmed the complexity of the current biological structure of the different human groups peopling the subcontinent, as demonstrated in earlier linguistic and anthropological studies. These studies highlight the history of this genetic complexity, but in the absence of fossil human remains it is still impossible to relate directly to the archaeological evidence, particularly with respect to the arrival of pastoralism.

Sheep and goats are of primary importance for past and present herders in southern Africa. In particular, different types of fat-tailed sheep/goats herded by the Cape Khoikhoi, by the Damara in northern Namibia and in many large modern farms in southern Africa. They are prized for their adaptability to climatic and environmental changes and arid conditions, which far exceed cattle in this respect. Their increased environmental resistance suggests that sheep may have reached the southernmost tip of Africa earlier than cattle (*Bos taurus*), which are more prone to diseases [77], [78]. However, since both cattle and sheep appear for the first time in northern Botswana during the same temporal span [17], [72], this region is often considered as a gateway for domesticates into southern Africa. The origin of the fat-tailed sheep is presently unknown and the skeletal remains discovered in archaeological contexts generally do not permit determinations of specific taxonomic levels (usually at family or even subfamily level), since the morphological distinction between sheep and goats is problematic in this region [72], [79]–[81]. In addition to comparative morphological analysis of all the archaeological remains of goat/sheep, genetic analysis of the Leopard Cave specimens (see [38], [78], [82]) will be undertaken, combining teeth, bones and dung from this and other Namibian sites, as well as the extant races of local caprines (the so-called “Damara goat” and “Damara sheep”).

Further excavation of Leopard Cave and other sites in Namibia, should confirm these preliminary results and continue to illuminate the major role that domesticates, especially sheep and goats, have played in the socio-economic and ecological adaptations of numerous population groups in southern Africa, both past and present.
